# Stakeholders’ perceptions on factors influencing male involvement in prevention of mother to child transmission of HIV services in Blantyre, Malawi

**DOI:** 10.1186/1471-2458-14-691

**Published:** 2014-07-07

**Authors:** Alinane Linda Nyondo, Angela Faith Chimwaza, Adamson Sinjani Muula

**Affiliations:** 1 School of Public Health, College of Medicine, University of Malawi, P/Bag 360, Blantyre, Malawi; 2 Kamuzu College of Nursing, University of Malawi, P.O Box 415, Blantyre, Malawi

## Abstract

**Background:**

Male Involvement (MI) in the Prevention of Mother to Child Transmission (PMTCT) of Human Immunodeficiency Virus (HIV) services is essential in a patriarchal society where men are decision makers of the household. Male partners have a role in the woman’s risk of acquiring HIV, uptake of HIV testing and participation in Mother to Child Transmission (MTCT) prevention programmes. Although MI is important for uptake of PMTCT interventions, it remains low in Africa. The purpose of this study was to identify factors that promote and hinder MI in PMTCT services in antenatal care (ANC) services in Blantyre, Malawi. Understanding of the factors that influence MI will assist in developing strategies that will involve men more in the programme thereby improving the uptake of PMTCT and HIV testing and counselling services by women and men respectively.

**Methods:**

An exploratory qualitative study was conducted from December 2012 to January 2013 at South Lunzu Health Centre (SLHC) in Blantyre, Malawi. It consisted of six face to face Key Informant Interviews (KIIs) with health care workers and four Focus Group discussions (FGDs) with 18 men and 17 pregnant women attending antenatal care at the clinic. The FGDs were divided according to sex and age. All FGDs and KIIs were digitally recorded and simultaneously transcribed and translated verbatim into English. Data were analysed using thematic content analysis.

**Results:**

Participants in both FGDs and KIIs identified the following barriers: lack of knowledge of MI in PMTCT, socioeconomic factors, relationship issues, timidity to be seen in a woman’s domain, unplanned and or extramarital pregnancies, fear of knowing one's HIV status, unwillingness to be associated with the service, health facility based factors, peer influence and cultural factors. The factors that would potentially promote male involvement were categorized into community, health facility and personal or family level factors.

**Conclusions:**

The factors that may hinder or promote MI arise from different sources. The success of MI lies on recognizing sources of barriers and averting them. Factors that promote MI need to be implemented at different levels of health care.

## Background

Male Involvement (MI) in the Prevention of Mother to Child Transmission (PMTCT) of Human Immunodeficiency Virus (HIV) services is essential in a patriarchal society where men are key decision makers as in most African countries [1-4]. A household head, who is usually a husband, greatly influences the woman’s ability to seek health care, implement health practices and interventions [5-7]. Male partners have a role in the woman’s risk of acquiring HIV [8-11], the uptake of HIV testing and interventions for reducing perinatal HIV transmission [8-12]. Success in the prevention of vertical transmission of HIV largely depends on the cooperation between partners [8] where a pregnant woman’s uptake of an HIV test is directly related to her husbands’ approval of the service [8,13,14]. Involvement of male partners in their partner’s PMTCT programme, in Tanzania and Kenya, led to more HIV infected women receiving Nevirapine during their antenatal follow up visits, avoidance of breastfeeding in their babies, adherence to infant feeding method chosen and a reported higher condom use than those whose partners were not involved [11,13].

Although there is positive impact on the uptake and adherence to PMTCT regimens especially when men accompany their partners, evidence indicates that only a few men accompany their female partners for antenatal care (ANC) and participate in PMTCT programmes [13] with rates of 3.2% in Malawi [15], 12.5% in Tanzania [11] and 16% in Kenya [16]. Efforts to involve men in ANC services, where PMTCT takes place, have only resulted in a few husbands being involved [13,17,18]. Prevention of Mother to Child Transmission services have been criticized for only focusing on females and sidelining males who are the primary support unit to the woman [19]. Barriers to MI in PMTCT services are categorized into: Health systems, community level and personal and family factors. Health system barriers include the clinic set up, using women to convey messages to men, service costs [20], distance to the clinic [21,22], and health workers’ attitudes [20,23]; Community level barriers are founded on traditional gender roles and cultural factors that regard pregnancy and its related aspects as solely a woman’s responsibility [2,11,20,24-26] and polygamous cultures that render MI challenging [27,28]. Personal and family level barriers may include the unwillingness and fear of a man knowing his HIV status [12,20,22,26], lack of knowledge [21-23,29-31], fear of stigma [22], a woman’s fear of violence [22,32] and socioeconomic factors [19,20].

Similarly, barriers to MI in PMTCT in Malawi have been categorized into personal and family and health system factors. Personal and family factors comprise unwillingness of men to know their HIV status [17], marriage instability [17], men’s lack of knowledge about the services [1], fear of potential dissolution of marriage consequent to HIV infected status [1,33], lack of perceived benefits of HIV services when one is healthy, lack of proper communication between partners and fear of stigmatization [34]. Barriers related to the health system were: health delivery system [35] such as services offered in an area traditionally viewed as a woman’s domain [17] and health centres being non-conducive for MI and cultural conformity to the traditionally gender defined roles [36].

Factors that promote MI in PMTCT are categorized at different levels as follows: personal and family, health facility and community. Personal and family level involves continuous discussions on MI at home and within the community [37]. Health facility level include: creating male-friendly environments [24], considering gender relations in the programme [38], offering adequate and private clinic space for male partners and accessibility of services [23]. Aligning clinic opening hours to men’s work schedule by opening over the weekends [11,19] or clinics that open for longer hours [4] and clinics that are geographically located closer to the targeted population [20] promotes MI. Streamlining of services for men or for pregnant couples only [39] through provision of services such as couple counseling [40-42] and sexual reproductive health services to boys and men [43] promotes MI. Even the term “prevention of Mother to Child Transmission” implies that responsibility of HIV transmission is solely on the mother. Activists and researchers have proposed a change on the term to “prevention of parents to child transmission” (PPTCT) to promote MI into the programme [44]. This proposition may benefit both the community and male partners.

Human resource related factors that would promote MI include providing ongoing education to the midwives, adequate staffing, improving the welfare of the health workers [20], empowering health care providers through precise policies and job descriptions on MI in PMTCT [23]. At community level, promotions that can be done include “mass campaigns” on couple HIV testing, use of key and influential people to promote MI [19,45], mass media campaigns such as television programmes [45] on MI, community mobilization [39] and involvement [26], male peers reaching to other men [46] and community based programme and support [26].

Male involvement, although it is important for uptake of PMTCT interventions, is low. In Malawi, male involvement is encouraged however there has been suboptimal involvement with self-reported rates by women ranging from 3.2% to 23% [15,36,47]. Understanding of the factors that influence MI may assist in developing strategies that will involve men better in the programme thereby improving the uptake of PMTCT services by women and may provide an HIV testing and treatment service for men. The main purpose of this study was to identify the factors that promote and those that hinder MI in PMTCT services in antenatal care services in Blantyre, Malawi.

## Methods

As part of a formative study, an exploratory qualitative study was conducted in Blantyre Malawi from December 2012 to January 2013 prior to an interventional study. A total of six Key informant Interviews (KIIs) and four Focus Group Discussions (FGDs) were conducted. These methods were selected based on their ability to offer a detailed expression of participant’s feelings and experiences from their own perspective. These methods also allowed for contextual understanding and definition of behaviours or events by different people [48]. The 6 KIIs provided diversity among the various health professionals at the health centre. The Health centre has Medical Assistants, HTC counsellors and Nurse Midwife Technicians. Thus, the 6 KIIs adequately represented the staff available. The 4 FGDs and 6 KIIs were selected because literature indicates that qualitative sample sizes are mostly small and usually less than 50 [49]. Potential participants who were unwilling to participate in the FGDs cited time constraints as the major reason. The RATS guidelines for reporting qualitative studies were followed in this study. (See Additional file 1 for the RATS checklist).

### Setting

The formative study was conducted in Blantyre district in the Southern part of Malawi at South Lunzu Health Centre. The reported HIV prevalence in 2011 among pregnant women in Blantyre was 10.4% [50]. The health centre was selected because of its semi urban setting.

### Selection and recruitment of participants

#### Female FGD participants

Female participants were conveniently sampled because of ease of access of potential study participants. Female participants for FGDs were recruited through the antenatal clinic following the eligibility criteria for the study. Potential discussants were approached by the researcher or research assistant in the waiting area before antenatal consultation who explained the purpose of the study. Inclusion criteria included the following a) pregnant women irrespective of HIV status and gestation dates, b) willingness to participate in FGDs, c) ability to give written consent and d) women with a male partner and e) above 18 years old. Eligible women were asked to remain after antenatal consultations for the discussions. Written consent was obtained from all study participants prior to participation.

#### Male FGD participants

Male participants were conveniently sampled and recruited with assistance from health care workers. Potential men were identified in the departments within the clinic and also some from the catchment area of SLHC. The men were booked for a discussion on a specific date and time at a preferred venue. The inclusion criteria for men were as follows: a) fathers with the youngest child below 5 years of age or have a wife who is currently pregnant. It was assumed that this group of men would have experienced PMTCT services by virtue of their wife being pregnant and would contribute to the discussions, b) willingness to participate in the FGDs c) ability to give written consent, d) above 18 years of age and men that were either using the health centre for medical care or not.

#### KII participant selection

Key informants were purposively selected according to their knowledge and responsibilities on PMTCT of HIV services. The choice for KII participants was secondary to their potential ability in offering a detailed description on factors that influence MI in PMTCT services [49]. Various health professionals were purposively sampled to achieve a diversity of professionals involved with PMTCT service implementation at SLHC to achieve a broader variety of responses. The interviews drew from the expertise of the informants in provision of PMTCT services.

Specifically, the Medical Assistant was selected because of her role in medical care provision to pregnant women and men at the clinic hence she provided more insight from the clinical and health system perspective. The Nurse Midwife Technicians were selected because they were the primary implementers of PMTCT services and were in close contact with pregnant women and their families. The HTC counsellors who are also Health Surveillance Assistants in their respective communities were selected based on their role in HIV testing and counselling of both women and men and their link between the clinic and the community. The Blantyre district PMTCT coordinator was selected because she coordinated all PMTCT activities in the district at the time of the study thus resourceful on the topic. The Nurses and counsellors from the Clinic were selected with assistance from the Nurse In charge at the Health Centre with a major criterion of providing PMTCT services. Following identification, the researcher individually scheduled appointments with the potential participants to conduct the interviews after obtaining informed consent.

### Data collection

A total of six KIIs were conducted with health care workers; two nurse midwife technicians, one medical assistant, two HIV Testing and Counselling counsellors and the PMTCT coordinator for Blantyre district. Each key informant attended a single interview which lasted for 45–75 minutes. The interviews followed a key informant interview guide (See Additional file 2) and the Principal Investigator conducted them. Additionally, during the same period, four FGDs were conducted with 17 pregnant women attending the antenatal clinic at SLHC and 18 men identified from the clinic and the catchment area. The FGDs groups were divided according to age and sex and translated into two male and two female FGDs which were as follows; a) younger female group with an age bracket of 18–24 years and it had 9 participants, b) older female group with an age bracket of 25 years and above and it had 8 participants, c) younger male group with an age bracket of 18–24 years and it had 8 participants and d) older male group with an age bracket of 25 years and above and it had 10 participants.

Male participants were randomly selected and were not partners to the female participants. The FGDs followed a focus group discussion guide (See Additional file 3). The duration of each FGD depended on the group interaction and ranged from 60–90 minutes. The PI facilitated the discussions with assistance from two study protocol trained research assistants in a private room within the health centre. All FGDs and KIIs were digitally recorded and then transcribed and translated verbatim into English concurrently by the PI. Research assistants checked the transcript for completeness and proof read the transcripts against the recorded interviews. Data collection stopped when the same responses were offered repeatedly in the various groups and among the groups.

### Ethical considerations

Ethical approval for the study was obtained from University of Malawi’s College of Medicine Research and Ethics Committee (COMREC, P.09/12/1279) while institutional permission to conduct the study was granted by Blantyre District Health Office (DHO). Written informed consent for participation and digital recording was obtained from each participant. Participation was voluntary and participants had the right to withdraw from the interviews and discussions at any point without any consequences. Participants were identified by codes for confidentiality purposes. Study documents were stored in locked cabinets. Pregnant women and men that declined participation in the study were assured that their decision would not compromise receipt of their regular medical care at the Health Centre.

### Data analysis

Data transcripts were exported to NVivo version 9.0 and were analysed using a thematic content approach. Thematic content analysis was selected because of its flexibility with qualitative data analysis secondary to its non-dependence on preexisting theoretical framework [51]. Some themes were deductively coded from the interview guides, conceptual framework and objectives of the study while some were processed inductively from the data transcriptions. Similar and recurring codes were later grouped under one theme. Initial coding was done by the PI and validated by another independent researcher. Interpretation of themes was realised by reading and rereading the transcripts as well as verifying with literature and colleagues. The developed themes were verified repeatedly against the digital recordings.

## Results

### Demographic characteristics of the study participants in the FGDs

Demographic characteristics of the participants in the FGDs (Table 1) were as follows: About half of the participants in the FGDs were males (n = 18). The largest tribal groups were Lomwes (14), 11 belonged to the Roman Catholic Church. Many participants, (n = 19) were educated to secondary school level as were their partners (n = 20) and half of the participants (n = 17) were self-employed while their partners were either formally employed or self-employed (n = 13 in each case). The median age for the female participants was 23 with a range of 18–41 years while for the male participants it was 26.5 with a range of 20–33 years. The median gravidity for the women was 2 with a range of 1–8 while the median age of youngest child for male participants 2 years old with a range of 1–6.

**Table 1:** Demographic characteristics of the study participants in the FGDs (N = 35)

**Variable**	**Male**	**Female**
Sex	18	17
Median age	26.5 (20–33)	23 (18–41)
**Religion**		
Roman catholic	4	7
Muslim	1	2
Seventh day adventist	3	0
Pentecostal	5	4
Other	5	4
**Tribe**		
Yao	2	5
Ngoni	3	4
Lomwe	8	6
Other	5	2
**Education level**		
Primary	5	9
Secondary	12	7
College	1	1
**Employment status**		
Not employed	0	14
Formal	3	1
Self employed	15	2
**Age of youngest child (Males only)**		
Pregnant wife	4	
Under 1	6	
1- < 2 years old	1	
2- < 3 years old	2	
3- < 4 years old	3	
4- < 5 years old	2	

### Demographic characteristics of the participants in the KIIs

Demographic characteristics of the six KIIs participants were as follows: five were females with one male. Four were married, one was single and one was widowed. In terms of religion, two were Presbyterians, two were Seventh Day Adventist members, one was a Baptist and one was a Roman Catholic. Half of the participants were Ngonis by tribe; one was Chewa, one Yao, and one Lomwe. Four participants were educated to college level while the remaining two were educated to secondary school level. Four of the respondents had worked for less than 5 years while one had worked for 6–10 years and another for 10–15 years. The median age for KII participants was 30.5 years with an age range of 24–42 years.

### Barriers to MI in PMTCT of HIV services

The pregnant women, men and health care workers interviewed described various factors that prevent MI in PMTCT of HIV services. These factors were categorized as follows; individual factors, socioeconomic, marital factors and health care system factors. Additional file 4 shows the distribution of the barriers according to the participants’ groups.

### Individual factors

#### Lack of knowledge

From the FGDs and the KIIs it emerged that in some instances men were not involved, or had limited involvement, in PMTCT of HIV services because of inadequate knowledge about the service and their role in the service. Respondents believed that men would be more involved if they were adequately informed about the existence of the service and had a clear definition of their role in the service.

“*There is need for education in our communities to understand the benefits of accompanying a partner for antenatal care, most of them (men) do not know the importance (of MI), such that even if their wives asked them to accompany her, they would not do it. They do not understand the importance or relevance of it”. KII respondent*

#### Timidity

Participants in both the younger males and females FGDs reported that some men shun PMTCT of HIV services because of their own shyness with women. Conversely, it was also expressed that some women prefer attending antenatal care alone because they would be embarrassed with their partner’s company.

“Some men are so shy to sit at the same place with women, the first day I started antenatal care my husband came, but he sat far off and when I asked him to come sit with me, he refused because he was shy”. Younger Women Focus Group (YW FG)

Timidity on the part of men may sometimes result from fear of their peers’ disapproving their choice of a partner as expressed in this quote:

“Some men are embarrassed because they may have showed their friends their partners and the friend must have commented that she is old …… so such things frustrate a man and he decides against being seen in the company of his wife to avoid embarrassment”. YM FG

#### Fear of knowing one’s HIV status

Participants from both the KIIs and FGDs reported that some men are not involved in PMTCT of HIV services because they are afraid of learning their HIV status directly or indirectly. It was reported that attendance to the service entailed taking an HIV test; therefore most men would opt for non-involvement in order for them to avoid the test.

“Sometimes, men refuse to come because they do not want the test (HIV test), because they know what they have been up to, they fear that they may likely be infected hence most of them refuse”. YW FG

In some instances, secondary to fear of learning one’s HIV status, some men regarded their wives’ HIV status as a proxy for them. This is illustrated in the following excerpt

“.......like for me, when I tested and when I told him that I had an HIV test and I am fine, I am uninfected, he said ahh if you are fine and since we are one body then if you are not infected then I am also not infected. So why should I have a test………” YW FG

#### Lack of interest or unwillingness

Female participants from FGDs and the KII participants felt that some men do not participate in PMTCT of HIV services because they are not interested or are unwilling to take part in the service. The lack of perceived benefits from the service by men coupled with men undermining the importance of the service to the family, emerged as reasons for men’s unwillingness to participate in PMTCT services. As such, a man would rarely seek permission from work in order for him to accompany his wife for antenatal care. Additionally, regarding work as a barrier to participation, male participants expressed the inability to obtain release from work. Other participants from female FGDs expressed that some women do not want partners to be involved, and in such cases a man will not be invited for ANC or be briefed on the need for his involvement.

“Some have no interest at all so they just take the issue lightly. They regard the whole thing (PMTCT Service) as irrelevant …….”. YW FGD

### Socio economic factors

#### Competing interests

The opinions of the FGD participants and KIIs were very similar on the dilemma that arises between the need to provide for the family versus the demand for involvement in PMTCT services. Participants expressed that although some men are willing to be involved; they fail to participate in the programme because of work or business obligations in order for them to provide for their families. However, the participants also suggested measures that can be put in place to overcome this barrier.

“For some of us casual laborers if we attend antenatal care we lose time and will not be able to find food so a man may not show up”. Younger Male Focus Group (YM FG)

Health care workers were aware that some men are not involved in PMTCT of HIV services secondary to socioeconomic factors. In light of that, health care workers have put measures such as fast and immediate attendance to pregnant women who attend ANC with their male partners so that such men attend to their work commitments afterwards.

#### Unusual requests

One female FGD participant narrated a scenario of how it may be awkward for a man to be excused from work because his superiors may consider it as a rare and peculiar request.

“For instance at his work place, a man goes to his boss and asks if he could be excused from his work temporarily as follows: “Boss I want to escort my wife to the hospital” the boss asks “what is she suffering from?” he responds “she wants to start her antenatal care or she wants to go to the maternity ward”. He will ask him of the validity of his request. Just with that question, he will think again over his request ….. When he gets back to his friends, they will ask him “what were you asking for?” He responds “I wanted some time out so that I escort my wife to the hospital and the boss laughed at me”; His friends will be puzzled and will ask him “Have you been fed some herbs “khuzumule” or what?” OW FG

### Marital factors

The nature of marital relationships between partners could be a barrier to MI in PMTCT services. Only male participants from FGDs regarded marital issues as a deterrent to their participation in PMTCT services. These were further categorized as follows:

#### Atmosphere within the family

Male participants from FGDs reported that the quality of the relationship between partners determines a man’s involvement in PMTCT of HIV Services. They believed that if a couple has outstanding problems and are continuously fighting over issues; that atmosphere would prevent MI in PMTCT services. The following quote illustrates this

“Sometimes if there are disagreements in a family, you may not have time to discuss on the need to get to the hospital together”. YM FG

#### Origins of the marriage

Male participants in FGDs reported that pre-arranged marriages would deter MI in PMTCT of HIV services because in such cases a man is not fully interested in his wife and consequentially he would not be involved in her antenatal care.

“I think some of the reasons (for lack of MI) are secondary to pre-arranged marriages. It is possible that a man did not want to get married to the specific lady however it was arranged that a man gets married to that lady ….., when she is pregnant this man will tell her to go on her own because he was forced to have her for a wife and did not want her such that one feels embarrassed to be seen with her”. Older Male Focus Group (OM FG)

#### Demanding or nagging wife

Male participants stated that some women are very demanding in relation to their needs while pregnant. The mood swings, demands and attitude that a woman displays while pregnant may cause a man to turn away from involvement. They expressed that a woman sometimes uses pregnancy as an excuse for getting all her needs met by her husband.

“*Some women are a burden “a cross” (mtanda) you try to find out her programme and even offer to accompany her on her next visit for antenatal care, she then gives you a budget exceeding the amount of money a man has. ………… this eventually prevents a man from accompanying his wife for ANC”. OM FG*

#### Unplanned pregnancies or extramarital pregnancies

Male participants expressed that unwanted pregnancies within marriage or through extramarital affairs would discourage their involvement in PMTCT of HIV services. They reiterated that in instances where a woman wanted a baby more than her husband; a man would rarely be involved because he was unprepared for that responsibility. Equally, a man with extramarital affairs, whose partners are pregnant, would rarely be involved in his partner’s antenatal care for fear of direct exposure of his extramarital affair which may lead into quarrels between his partners. This was shown in the following quote:

“Sometimes, some of us men have extramarital affairs, we may have a wife but also have girlfriends so sometimes one is afraid that if he shows up at the clinic there could be fights when the wife and the girlfriend meet, so it is better for a man to wait under a tree and let the woman go on her own for antenatal care”. OM FG

### Health care system factors

Study participants reported health care system barriers. The factors could further be divided into two categories i) health facility barriers which includes a) availability of supplies, b) facility set up, and c) nature of services; and ii) Health provider barriers which includes a) lack of professionalism and b) inadequate number of health care providers.

### Health facility factors

#### Availability of supplies at the health care facility

Stock out of resources such as HIV test kits prevents men from being involved. Since HIV testing is the entry point to PMTCT services, participants from one FGD reported that some men are discouraged to attend to PMTCT of HIV services because of the erratic availability of supplies such as test kits. The following quote illustrates this:

“…… on this particular day he was absent from work and accompanied me for antenatal care, and we learnt that there were no testing kits… because on that day he did not report to work, he just said, he will never come again and has never done so again”. YW FG

#### Facility set up

The lack of privacy in the layout of a facility is a barrier to MI in PMTCT of HIV services. The FGDs and KIIs participants reported that the clinic did not have adequate space for all services that it offers. As a result there were three to four services being offered in proximity to each other. In view of that proximity, some participants stated that it would be uncomfortable for one to be in a conspicuous setting where one may be easily seen by other people thereby compromising one’s privacy.

“…. at South Lunzu Health Centre …….. the section where the maternity wing is located, where pregnant women are counseled, it is a place that is very close to the place where other people consult the clinician, receive medication, the door is the same, and again there is no privacy (wall) at this place, some men are uncomfortable with that, whereas if there was private space most men will be free to be involved …….”. YM FG

#### Nature of services

One health worker in the KIIs stated that the messages that are sung at the antenatal clinic discourage men from being involved. Messages in some songs exclude men and only target the women.

“Even the songs we always sing at the antenatal clinic, we always mention the woman, not a man, so the men feel ashamed to be together with their wives, they feel that they are not part of it”. KII Respondent

### Health care worker factors

#### Lack of professionalism

Participants in the male FGDs and KIIs reported that lack of perceived professionalism and unwelcoming attitude of health care workers as barriers to MI. Human Immunodeficiency Virus services require sensitivity thus involves higher levels of confidentiality and privacy which is not currently the case. Participants stated that some health care workers may not keep HIV test results in confidence.

#### Inadequate health workers

The health care providers reported shortage of health workers as another barrier for MI. The health care providers unanimously agreed that MI in PMTCT of HIV services is important and a welcome service however implementing strategies for MI with the current level of staffing has potential challenges.

“You know one of it can be the shortage (shortage of staff) …. for these men to be attended to, it requires a nurse dedicated to couples while another nurse attends to women who have come without a male partner……”. KII Respondent

### Cultural/gender factors

#### Woman’s domain

Culture and gender considerations were perceived as a barrier. Participants from both the FGDs and KIIs reported that culturally, secondary to division of roles according to gender, pregnancy and child bearing issues are a woman’s responsibility and that men are partially involved. The cultural beliefs regarding pregnancy therefore make it difficult for men to be involved.

“It appears to be “zachizimayizimayi ehe” only for women such that for a man to be among a group of women and discuss women’s issues, it seems as if a man is wasting his time”. OM FGD

#### Man as the head of house notion

Rooted in the hegemonic notion of masculinity, particularly from the male FGDs, it emerged that men are heads of the household and command respect. Men strongly believed that as household heads, they are decision makers in the family. This notion prevents them from attending to PMTCT of HIV services because these services are targeted towards women thereby undermining his position and masculinity if he attends. This is reflected by the following quote

“One actually knows that what his wife is saying is important (about MI in PMTCT) and what she is suggesting (that he participates in PMTCT) is the right thing but for a man as the head of the family, he may not accept that, he fears that he will lose his authority and his wife will now be controlling him”. OM FGD

#### Sub category: Khuzumule

An interesting category emerged, and sparked vibrant discussion in both the male and female FGDs. It was repeatedly observed that a man, who heeds his wife’s request, even if it is a request to accompany her for PMTCT of HIV services, will be considered to have unknowingly taken a traditional herb called “Khuzumule”. It was common belief that following ingestion of “khuzumule” a man will do as the wife pleases and a man who is seen around the antenatal clinic and involved at every stage would be considered to have taken a portion of that.

### Factors that promote male involvement in PMTCT of HIV services

In response to a question on factors that may promote male involvement, the respondents suggested several ways in which MI in PMTCT of HIV services may be promoted or enhanced. Promotions could be at different levels for instance a) Community, b) Health Facility and c) Personal. Additional file 5 shows the distribution of the promoting factors according to the participants’ groups.

### Community level factors

#### Community sensitization

Taking advantage of community events as they occur in the community may promote MI in PMTCT of HIV services. Participants proposed that during community events or open-day functions, messages on MI in PMTCT of HIV services may be shared at that forum. Other messages could be in form of posters which would spark interest in men eventually leading to their involvement. Currently, posters are available at the health centre but not in the communities.

“….. there can be a function in the community and other people will be drawn to the activities and because the message on male involvement is embedded within the activity then they get to know about the message there”. YM FGD

Key informants suggested that health workers should proactively reach out to the community and sensitize them, especially men, on the relevance of MI in PMTCT of HIV services and how they can be involved.

#### Involvement of chiefs and other influential people

Key informants stated that involving chiefs and other influential people in the programme would promote MI in the services. Chiefs are custodians of culture and enforcers of customs and activities in communities where formal health services are situated. In light of their position and influence, they would ensure that their community adheres to what has been recommended by the health care workers.

“I think mostly, involvement should begin at different levels, we can use the chiefs, the church, the groups in which we belong in, we can inform the couple the importance of them coming together. Sensitization should start from the villages so that when they report here it will be like a continuation of what started out there”. KII Respondent

### Health facility level factors

#### Health education sessions

Participants suggested inclusion of MI in PMTCT as part of the health education that take place in the outpatient department, family planning clinic of the health facility in order to reach more people. The health education on MI should not be limited to the antenatal clinic because most men patronize the General Medical Outpatient department than the antenatal clinic.

“Health talks within the OPD tackling on male involvement, because if we only discuss it in antenatal clinic we will only inform women only however in the OPD department the message will get to men as well”. KII Respondent

Within the health education, there ought to be clarification of the term “PMTCT”. A key informant advocated for precise explanation on the meaning of PMTCT to men. In as much as the term refers to “mother” it should be emphasized to men that such a reference does not exempt them from the services.

“And even for the name of PMTCT, it should be explained in detail so that the woman should not be blamed in instances where both the woman and her baby are HIV infected …….”. KII respondent

Additionally, participants believed that offering explanations to men on the procedures and events that take place at the antenatal clinic will encourage men to be involved in PMTCT HIV services.

“This man needs to be adequately informed so that he fully understands on how he can take part in protecting his family, some men lack knowledge on the issues ……they may not have enough opportunity to learn more on these issues”. OM FGD

The older women in the FGDs believed that with proper and adequate explanations on ANC procedures, men that are employed will be able to excuse themselves from work.

#### Male friendly environment

Key informants expressed the relevance of a male friendly environment such as private room that can be used for couples counseling and interventions on PMTCT. This environment may eliminate shyness which also influences MI. This was described in the following ways:

“ some men are shy, ………. so if we could have a private room for the men to use, for them to be attended to faster, that would be nice”. KII Respondent

Additionally, it was suggested that integration of services would promote MI, such as men having their other health needs attended to holistically within PMTCT of HIV services.

#### Clinic flow management

Key informants asserted that ensuring that couples that come together for PMTCT of HIV services get preferential treatment, would promote MI in the service. They were cognizant that some men have work commitments therefore will need to be released early for such responsibilities.

“………..a pregnant woman who has come with her partner must be assisted first, being respectful to them, maintaining privacy. Then most men would come because they will know that they will not take long, the discussion will be in private because there will be the nurse and my wife”. KII respondent

#### Reward based promotions

Key informants suggested the use of a reward based system in order to promote MI in PMTCT of HIV services. It was suggested that a woman who attends the service with her partner should get a reward such as a *chitenje* (cloth wrap) or T-shirts.

#### Initial antenatal visit

Female participants in FGDs stated that attendance to the initial antenatal visit as a couple should be implemented as a policy.

“For instance, the day when one starts antenatal clinic, they ask us to have an HIV test and get our results, what is needed is that on this day as we get the HIV test, it is important to be together, so that he is tested as well so that he also knows whether he is fine or not. We need to try our best and tell them that “as for me, I will not go for antenatal clinic because the requirement is that when one goes there, one should go with her husband to be tested for HIV together”. YW FGD

#### Attitude of health care workers

Key informants suggested that a positive attitude by health care workers as they render care to couples would promote MI in PMTCT of HIV services.

### Personal/family level promotions

#### Men to men promotions

In order to encourage men that are not patronizing the service, key informants and male FGD participants suggested use of men who are involved to reach other men, on an individual basis or through men only groups, and educate them on the relevance of their involvement in PMTCT services.

#### Upbringing of children

A Key informant stated that MI ought to start at a family level when children are young so that children grow up without separation of roles based on gender. It was suggested that children raised without gender definition of roles will not shun services that are traditionally regarded as women’s only responsibilities.

“….. however mostly it needs to start when someone is young, as a parent, one should not separate the children’s roles and responsibilities according to gender so that when they are growing up, so that in future there will not be separation of roles which will help them in future”. KII respondent

## Discussion

The main findings of this study on factors that influence MI in PMTCT show that the factors are interrelated among and within individual, community and health facility factors, such that a successful MI programme requires a multifaceted and multilevel approach that includes all the factors involved. The current study findings validate and augment on what has been reported by other studies in this area.

The individual factors that hinder MI as highlighted in this study remain consistent with other studies. A man’s lack of knowledge on the relevance and his role in PMTCT services limits his participation [17]. Additionally, lack of knowledge on pregnancy and its associated factors contributed to lack of MI in the PMTCT programme [2,17,22,23,52,53]. The lack of information also included men being unaware of the; existence of PMTCT services, the benefits and the role of a man in PMTCT services [23], not knowing the wife’s HIV status [54] and rationale for testing when they had no signs of sickness [55]. Low formal education in men further limits a man’s understanding of issues on HIV and AIDS [54]. As reported in other studies, due to inadequate information on HIV and AIDS, men have used their wives HIV test results as a proxy for their own HIV status [28,39,56]. Conversely, educating men in antenatal care aspects yielded positive results in birth preparedness and postnatal visit compliance [29].

The fear of learning one’s HIV status following attendance of PMTCT services prevents men from attending the service as has been reported by other studies [17,23,28]. In Lilongwe Malawi, lack of MI in PMTCT was reported to arise from men’s fear of HIV testing [57] while in Zimbabwe men perceived HIV as a threat to their manhood and they discouraged their partners in accessing ART services to avoid learning their HIV status indirectly [26]. Equally, women have also expressed the fear of learning their HIV status as a barrier to their participation in PMTCT programme [58]. However with the current opt out policy of HIV testing; women are rarely refusing an HIV test in Malawi as evidenced by the high rates of HIV testing antenatally [59].

A man’s lack of knowledge on PMTCT issues [55], his fear of learning his HIV status fear [17], and traditional gender roles and cultural norms [2,60] partially explain the unwillingness of men or lack of interest by men with the PMTCT programmme as expressed in this study, thereby, hindering their involvement. These results are congruent with reports from Tanzania where 74.6% of men were unwilling to participate in PMTCT programs [61] while in Zambia men had low motivation for MI [52,54] and others were uninterested in the service [62]. This study also showed that some women are unwilling to have their partners involved in ANC, probably because they want to retain the decision making and control on their reproductive health [62] or they may not have a male partner.

Timidity with involvement in a domain that is traditionally regarded as a woman’s responsibility was highlighted as a barrier in this study which remains congruent with other studies [2,28,39]. Similarly reviews of studies have reported that fear of societal stigma and ridicule [63,64] as barriers. Timidity may stem from intrinsic factors or be perpetuated by traditional mindset [54] as well as community beliefs [28,65] and is further compounded with the term “PMTCT” which excludes men in the program [19]. Timidity may also be aggravated by the manner in which services are rendered for instance in this study the songs sung in the antenatal clinic deter men from involvement. This resonates with findings by Kang’ona in Lilongwe, Malawi where men shunned PMTCT services because they were embarrassed to sing along at the antenatal clinics with their partners [57]. Timidity may also stem from notions of masculinity that propel the supremacy of a man.

The belief that a man is the head of the family who may not be influenced by his partner deters them from involvement in PMTCT services especially when invites come through his partner. This finding remains consistent with Tanzanian and Ugandan studies where women could not ask their partners for HIV tests because they had no authority over them [39,55] and was also concluded as a limiting factor for MI in maternal health services in Mwanza, Malawi [66]. Superiority norms held by men led to men shunning of any HIV related clinics for fear of being regarded as weak [65] or less masculine [39]. Furthermore in this study, participants regarded a man who follows what his wife tells him, to have unknowingly taken a local herb called “Khuzumule” which renders him a “puppet”. A similar nomenclature regarding men who are involved in pregnancy and its associated aspects has been previously reported as follows: in Lilongwe, Malawi such men were regarded as fools [57], in Nepal, such men were regarded as “*joitingre*” or “hen-pecked” [22] and in India they were regarded as “sissies” [62]. Furthermore, in Tanzania men stated that it was against their culture to be involved in female affairs [61]. It could be argued that for a successful MI in PMTCT service; cultural aspects, gender roles and dynamics of marital relationship need to be explored and incorporated in the development and implementation of the programme [62]. Conversely, a study by Tshibumbu in Zambia found that men did not regard, a man who escorted his partner for PMTCT services as bewitched [27]. We argue that as a head of the family a man should take responsibility and take a leading role in the health of his family.

Time constraints, such as balancing the need to provide for the family versus attendance to antenatal clinic and negotiating time off from work, is a barrier to MI in this study. This builds upon findings from other studies that identified socioeconomic demands [20], poverty [52] and job responsibilities [13,19,22,23,28,39,53,54,62,67] as a barrier to MI in PMTCT. Additionally, other studies on MI in sexual reproductive health reported that men prioritized social obligations [2] and other personal issues [54,61] than supporting their partners’ attendance to antenatal care. Time constraints also prevented men from listening to radio or reading brochure messages on MI in PMTCT [54]. As a way of averting this problem, countries may consider legitimizing MI in PMTCT for men that are formally employed while those in informal employment may utilize their free days to attend to the service.

Health system related factors highlighted in this study are similar with those reported in earlier studies such as services located in an antenatal clinic, non-male friendly environment there by marginalizing men [54,62,68,69], organization of the PMTCT programme [39], lack of supportive hospital policies on MI, and inadequate space [20,22,62].

According to our study, as also shown in others, human resource related barriers such as health care workers negative attitude [20,53,54,61] and shortage of health care workers [22,55] are a barrier to MI. Our findings remain consistent with a study in Lilongwe, Malawi that reported that men failed to participate in PMTCT services because of the health care workers’ rudeness [57]. However in our study, health care attitudes were reported as barriers by health care workers only. Nonetheless, health care workers’ attitude has been cited by women as a barrier for PMTCT services in Botswana [58]. Contrary to other studies [53,61] the fact that the majority of health care staff involved in PMTCT are females was not a barrier in this study. We argue that perhaps most men in this setting have not attended PMTCT services therefore they have no reference point or because traditionally the majority of health care workers in Malawi are females hence men are used to being attended to by women. Another factor that was not cited in the present study is the non-flexible opening hours [19,61] which has been cited by several studies as a barrier to MI. This may possibly be secondary to non-involvement as most men have not encountered it as a problem but it may also portray the flexibility of men with time. Furthermore distance [19,20,55] and cost of getting to the health centre as reported in other studies [19,55] was not mentioned as a direct barrier in this study.

### Promoting factors

Incentivizing couples that report together for PMTCT services is a promoting factor for MI reported in this study. Incentives, a part of behavioural economics, have a potential in increasing uptake and retention in PMTCT programmes [70]. This strategy, although reported in Mwanza Malawi, was regarded as unsustainable as it was dependent on donor funded incentives [66]. Nonetheless, in a PMTCT programme in Lilongwe Malawi, retention and appointment keeping were linked to nutrition and hygiene incentives that mothers received [71]. Other forms of incentives may be provision of transport to a PMTCT centre [55].

Having an all-male clinic or a pregnant couple clinic was suggested as a promoting factor for MI as also suggested in earlier studies [11,23,39,54]. Ensuring that a couple attends the initial antenatal visit together partially remains consistent with studies that have advocated for couple attendance to VCT/PMTCT or ANC [54]. The difference in this study is that participants only emphasized male attendance on the initial visit alone as opposed to all antenatal visits as they were cognizant of the socioeconomic demands on the man and most participants felt that major activities on PMTCT are covered on the initial visit. Making antenatal care attendance by men obligatory is a way of ensuring greater rates of MI in maternal health services in general [72]. This recommendation promotes the aspect of couple counselling which has been recommended by several studies [13,62] as a way of eliminating women only ANC and PMTCT services [62].

Creation of a male friendly environment within the antenatal clinics would promote MI. This finding remains consistent with earlier findings on ensuring a male friendly environment [73]. offering privacy [11,23,53-55,74] and an environment that handles men’s sexual and reproductive health matters [2]. A male peer approach as stated in this study was also advocated in PMTCT services [17,19,55] and other maternal health services [53,55,66]. Additionally, this approach would be culturally appropriate as men will be advised by fellow men [68] as opposed to being advised by women. Furthermore, this approach would offer a live personal communication which is deemed beneficial for increased understanding in men [55]. In a program in Lilongwe, Malawi, peer education through drama and male friendly hospital infrastructures were recommended as a way of promoting MI in PMTCT [75].

Clarification and education on the meaning and importance of PMTCT coupled with a change in name from PMTCT to Prevention of Parental transmission of HIV to child (PPTCT) has potential in promoting MI in the service. This finding is consistent with studies that showed that educating men on their role and relevance to PMTCT and including them in the programme would increase their involvement [17,19,20,23,27,76] and further supports what Msellati recommended on changing the term PMTCT to PPTCT as a way of accommodating men in the programme [44]. Another study proposed a change from voluntary testing to routine testing so that most couples get tested [77]. Currently in Malawi, as in most countries, Voluntary Counselling and Testing (VCT) changed to HIV Testing and Counselling (HTC).

Training of health care workers on MI in PMTCT services and incorporation of it in their job description and policies has potential in promoting MI. Similarly other studies have suggested training of health care workers at all levels [2,53-55] sensitizing employers and having a PMTCT friendly work policy [20,54,74]. We propose development of a MI in PMTCT policy in Malawi. Additionally, improving the attitude of health care workers and instilling maintenance of confidentiality as expressed in this study remains consistent with findings from other studies [54,55] that proposed integration of services as a measure of ensuring confidentiality [54].

Extending invites to men as expressed in other studies is also a way of promoting MI [20,23,53]. A review of literature concluded that MI requires a multimethod approach such as invitation letter accompanied by community education and mass media campaigns [78]. Ensuring consistent supply of resources remains consistent with a study by Mohlala that reported availability of ARVs as a promoting factor [79] for MI.

Community sensitization via open day functions, use of posters and use of influential people such as chiefs were suggested in this study as promoting factors. These findings remain consistent with other studies that suggested community mobilization [39,79] to enhance MI in PMTCT as well as use of community leaders through community outreach [74], public meetings in places like churches, bars or shebeens as strategies for promoting MI [53-55,66]. Use of community support structures in PMTCT services yielded an increase in HTC with improved outcomes in HIV exposed infants [80]. Community meetings have the potential of increasing uptake of couple counselling in PMTCT than radio messages because they provide a dialogue and men may have all their concerns addressed [55]. This strategy needs to be explored with MI in PMTCT. A review of Demographic Health surveillance data from 8 countries in Africa, recommended the recognition of an association between uptake of HIV tests by men and the communities they live in thereby underscoring the importance of community factors such as educating communities in HIV programmes to increase male uptake of the programme [81]. Another aspect of community mobilization for MI in PMTCT would be removing the gender differences that are embedded in raising children so that both boys and girls are raised without gender role boundaries which remain partially consistent with earlier findings MI [62]. This suggestion needs to be cautiously taken because it may only work in selected roles. It could be argued that changing the upbringing of children will entail a change in the cultural pillars and gender norms which have been highlighted in this study as barriers. It also has the potential of eliminating timidity and embarrassment which were also expressed as barriers in this study.

Use of posters and radio messages as suggested in this study, remains consistent with findings from Khayelitsha South Africa [53]. Additionally, TV programmes have been known to stir up conversation on HIV and AIDS between partners [62]. A combination of clinic based health education, use of radio messages and television has potential in positively influencing women’s intentions with HIV testing [82].

Other factors that promote MI that were not suggested by participants in this study were as follows: flexible opening hours [4,23,53], and newspaper messages on relevance of MI [53], possibly because of the population under study rarely has access to newspapers. Perceived benefits such as HIV free children [19] were not cited as a motivator for MI in the current study; we are therefore not sure, of the communities understanding on the benefits and aim of PMTCT interventions.

### Strengths

The strength with the study is the inclusion of responses from health care workers, men and pregnant women’s perception on the area under study. The information gathered in this study, presents consolidated reflections and views on the topic and represent men’s’ views better because of their involvement in the study.

### Limitations

Use of FGDs may not have allowed other group members to verbalize all their concerns however the researcher encouraged all members to talk and assured them of confidentiality. Although the researcher does not work at SLHC, holding the discussions at the clinic could have resulted in other participants not fully expressing themselves or giving what they deemed as socially desirable responses. This was minimized by defining the groups according to gender and age groups to ensure that people of the same gender and within the same age range are in one group. The use of convenience sampling, though common in qualitative research, coupled with the small sample size limits result generalizations beyond the research site, however they provide information that can be explored further. Our study did not rank the barriers and facilitators in order of priority thereby posing a challenge with prioritizing interventions.

## Conclusions

There are several factors that may hinder or promote MI. The success of MI depends on implementing interventions that will minimize the barriers whilst enhancing the factors that promote MI in a contextualized manner. Approaches will vary depending on the difference in cultures, couples and different health systems. At a health facility level, health care personnel need to proactively involve males by eliminating all the health care related factors whilst optimizing health care promoting factors. Other innovative measures need to be explored within the health system such as active couple counselling, pregnant couple clinics and extending working hours to accommodate working men. On a personal level, men need to be well informed on HIV for them to make informed decisions about their involvement. At community level, community leaders ought to encourage men in the various forms of communication and also amend cultural norms to accommodate MI. Involvement of chiefs is paramount for the success of the program. There is need for more gender and cultural studies with different cultures in order to understand how culture affects MI in PMTCT services.

## Competing interests

The authors declare that they have no competing interests.

## Authors’ contributions

ALN planned the study, developed study methods, interview guides and conducted the FGDs and KII, developed analysis plan, analysed the data and drafted the manuscript. ASM and AFC supervised the planning development of the methods, analysis plan, and data analysis and contributed and supervised the manuscript writing. All authors read and approved the final manuscript.

## Pre-publication history

The pre-publication history for this paper can be accessed here:

http://www.biomedcentral.com/1471-2458/14/691/prepub

## Supplementary Material

Additional file 1 RATS checklist. Click here for file

Additional file 2 Interview Guide for Key Informant Interviews. Click here for file

Additional file 3 Focus Group Discussion Guide. Click here for file

Additional file 4 Distribution of Barriers to MI in PMTCT among Men, Women and Health Care Workers. Click here for file

Additional file 5 Distribution of Promoting Factors for MI in PMTCT among Men, Women and Health Care. Click here for file
