# Supplementary material for: Stakeholders’ perceptions on factors influencing male involvement in prevention of mother to child transmission of HIV services in Blantyre, Malawi
Source: BMC Public Health. 2014 Jul 7;14:691. doi: 10.1186/1471-2458-14-691 (PMC4226974; doi:10.1186/1471-2458-14-691)
Supplement: Additional file 2 — Interview Guide for Key Informant Interviews. [file 1471-2458-14-691-S2.docx]

**Introduction**

I would like to thank you all for coming today. My name is Alinane Nyondo. I am a student at College of Medicine in Blantyre.

I am conducting KII with Health Care workers at SLHC and Blantyre DHO as part of a project on MI in PMTCT. Your opinions are very valuable to me.

**Informed Consent Section**

Before we start the discussions, I would like to ask for your written informed consent following the Informed consent form that describes the study in detail.

*Note: Informed consent will be obtained following the ICF*

*Socio demographic details will be collected as per socio demographic Questionnaire for KII.*

**Discussion**

Before we start the discussions please introduce yourself by telling me your nature of work and what you like most about your work.

How long have you been involved in provision of PMTCT services?

Role at Health Centre_______________________

**Interview Session**

**Objective 1- Description of MI in PMTCT**

1. Would you please describe Male Involvement in Prevention of Mother to Child Transmission of HIV (MI in PMTCT) services in your own terms?
2. What is the relevance of MI in PMTCT services?
3. Describe the current level and type of MI in PMTCT?
4. What would be regarded as the desired level or type of MI in PMTCT?

**Objective 2- Barriers to MI in PMTCT**

1. What are some factors that make it difficult for MI in PMTCT Services?
2. What are the challenges with MI in PMTCT?

**Objective 3 and 5- Factors that promote or enhance MI in PMTCT**

1. What are some factors that would promote or facilitate MI in PMTCT?
2. How many men roughly accompany their partners for PMTCT services?
3. What kind of involvement do these men display?
4. How does culture and gender influence MI in PMTCT Services?

**Objective 4- Strategies for MI in PMTCT**

1. What are some factors that would encourage a male partner to be more involved in PMTCT services?
2. What are the resources that are needed in order to promote MI in PMTCT?
3. What are some of the strategies that may be used to promote MI in PMTCT services?
4. Out of the strategies outlined, which one would be the best strategy to use and try out now?
