# Supplementary material for: Stakeholders’ perceptions on factors influencing male involvement in prevention of mother to child transmission of HIV services in Blantyre, Malawi
Source: BMC Public Health. 2014 Jul 7;14:691. doi: 10.1186/1471-2458-14-691 (PMC4226974; doi:10.1186/1471-2458-14-691)
Supplement: Additional file 3 — Focus Group Discussion Guide. [file 1471-2458-14-691-S3.docx]

**Introduction**

I would like to thank you all for coming today. My name is Alinane Nyondo and my assistant is ________________________. I am a student at College of Medicine in Blantyre.

Our research team will be conducting FGD with men and women within this area as part of a project on MI in PMTCT. By PMTCT we are referring to services that are provided to limit transmission of HIV from mother to child. These services include HIV Pretest t and Posttest counseling, HIV testing, if infected provision of ART for PMTCT, Infant feeding options. We feel the services may be improved if we incorporate views of men and women. Your opinions are very valuable to us

**Informed Consent Section**

Before we start the discussions, we would like to ask for written informed consent following the Informed consent form that describes the study in detail.

*Note: Informed consent will be obtained following the ICF*

Collect Socio demographic details as per socio demographic Questionnaire.

**Discussion**

Before we start the discussions please introduce yourself by telling us the number of children you have, whether you are currently working and the nature of work

In your communities, how many children do people usually have?

**Objective one- description of MI in PMTCT**

1. Would you please describe Male Involvement in Prevention of Mother to Child Transmission of HIV (MI in PMTCT) services in your own terms?
2. What is the relevance of MI in PMTCT services?
3. Describe the current level and type of MI in PMTCT?
4. What would be regarded as the desired level or type of MI in PMTCT?

**Objective 2-Factors that affect MI in PMTCT**

1. What are some factors that make it difficult for MI in PMTCT Services?
2. What are the challenges with MI in PMTCT?

**Objective 3 and 5- Factors that promote MI in PMTCT**

1. What are some factors that would promote or facilitate MI in PMTCT?
2. How many men roughly accompany their partners for PMTCT services?
3. What kind of involvement do these men display?
4. How does culture and gender influence MI in PMTCT Services?

**Objective 4- Strategies for MI in PMTCT**

1. What are some factors that would encourage a male partner to be more involved in PMTCT services?
2. What are the resources that are needed in order to promote MI in PMTCT?
3. What are some of the strategies that may be used to promote MI in PMTCT services?
4. Out of the strategies outlined, which one would be the best strategy to use and try out now?

**Conclusion**

We are now reaching the end of the discussion. Does anyone have additional comments before we conclude the session? Thank you very much for your participation in the discussion, your opinions are valuable for MI in PMTCT.
