# Supplementary material for: Stakeholders’ perceptions on factors influencing male involvement in prevention of mother to child transmission of HIV services in Blantyre, Malawi
Source: BMC Public Health. 2014 Jul 7;14:691. doi: 10.1186/1471-2458-14-691 (PMC4226974; doi:10.1186/1471-2458-14-691)
Supplement: Additional file 4 — Distribution of Barriers to MI in PMTCT among Men, Women and Health Care Workers. [file 1471-2458-14-691-S4.docx]

| **Barriers** | **Men** | **Women** | **Health Care Workers** |
| --- | --- | --- | --- |
| 1. **Individual Barriers** |  |  |  |
| *Lack of Knowledge* | *** | *** | *** |
| *Timidity* | *** | *** | *-* |
| *Fear of Knowing one’s HIV status* | *** | *** | *** |
| *Unwillingness or lack of interest* | *-* | *** | *** |
| 1. **Socio economic Barriers** |  |  |  |
| *Competing interests* | *** | *** | *** |
| *Unusual request* | *-* | *** | *-* |
| 1. **Marital Factors** |  |  |  |
| *Atmosphere within marriage* | *** | *-* | *-* |
| *Origins of the marriage* | *** | *-* | *-* |
| *Demanding or Nagging wife* | *** | *-* | *-* |
| *Unplanned pregnancy or Extra Marital affairs* | *** | *-* | *-* |
| 1. **Health Care Factors** |  |  |  |
| 1. **Health Facility Factors** |  |  |  |
| *Availability of resources* | *-* | *** | *-* |
| *Facility set up* | *** | *-* | *** |
| *Nature of services* | *-* | *-* | *** |
| 1. **Health Care worker Factors** |  |  |  |
| *Lack of professionalism* | *** | *-* | *** |
| *Inadequate Personnel* | *-* | *-* | *** |
| 1. **Cultural and Gender Factors** |  |  |  |
| *Woman’s domain* | *** | *** | *** |
| *Head of the house* | *** | *-* | *-* |
| *“Khuzumule”* | *** | *** | *-* |

**Additional File 4: Distribution of Barriers to MI in PMTCT among Men, Women and Health Care Workers**

*** = expressed it as a barrier;**

**-= did not express it as a barrier**
