# Supplementary material for: Stakeholders’ perceptions on factors influencing male involvement in prevention of mother to child transmission of HIV services in Blantyre, Malawi
Source: BMC Public Health. 2014 Jul 7;14:691. doi: 10.1186/1471-2458-14-691 (PMC4226974; doi:10.1186/1471-2458-14-691)
Supplement: Additional file 5 — Distribution of Promoting Factors for MI in PMTCT among Men, Women and Health Care. [file 1471-2458-14-691-S5.docx]

| **Promoting Factors** | **Men** | **Women** | **Health Care Workers** |
| --- | --- | --- | --- |
| 1. **Community Level** |  |  |  |
| *Community sensitization* | *** | *** | *** |
| *Using Chiefs or influential People* | *-* | *-* | *** |
| 1. **Health Facility Level** |  |  |  |
| *Motivation Talks* | *** | *** | *** |
| *Male Friendly environment* | *-* | *-* | *** |
| *Improving Clinic Flow* | *-* | *-* | *** |
| *Reward Based promotions* | *-* | *-* | *** |
| *Initial Antenatal Visit* | *-* | *** | *-* |
| *Attitude of health care workers* | *-* | *-* | *** |
| 1. **Personal and Family Level** |  |  |  |
| *Men to men promotions* | *** | *-* | *** |
| *Upbringing of children* | *-* | *-* | *** |

**Additional File 5: Distribution of Promoting Factors for MI in PMTCT among Men, Women and Health Care**

*** = expressed it as a promoting factor;**

**-= did not express it as a promoting factor**
